# Recurrent Occupational Hantavirus Infections Linked to Feeder Rodent Breeding Farm, Taiwan, 2022

**DOI:** 10.3201/eid3008.230875

**Published:** 2024-08

**Authors:** Kung-Ching Wang, Chih-Kai Chang, Shu-Fen Chang, Pei-Yun Shu, Hsi-Chieh Wang, Shin-Wei Su, Fang-Ling Lin, Chung-Yu Wang, Chia-ping Su

**Affiliations:** Taiwan Centers for Disease Control, Ministry of Health and Welfare, Taipei, Taiwan (K.-C. Wang, C.-K. Chang, S.-F. Chang, P.-Y. Shu, C.-p. Su);; Institute of Environmental and Occupational Health Sciences, National Taiwan University (H.-C. Wang, S.-W. Su, F.-L. Lin, C.-Y. Wang);; National Tsing Hua University School of Medicine, Hsinchu, Taiwan (C.-p. Su)

**Keywords:** hantaviruses, viruses, zoonoses, occupational diseases, feeder rodent farms, Taiwan

## Abstract

We investigated 2 acute cases and 1 previous case of Seoul hantavirus infection in workers in a feeder rodent breeding farm in Taiwan. Prevalence of hantavirus IgG among the tested feeder rats was 37.5%. Appropriate prevention measures, including using disinfection protocols and personal protective equipment, are crucial to lowering risk.

Each year in Taiwan, hantaviruses cause 0–4 human cases of hemorrhagic fever with renal syndrome (HFRS) (*1*). *Rattus norvegicus* rats are a notable local reservoir host for Seoul virus (SEOV), a hantavirus causing HFRS, but SEOV also is found in other rat species, such as *Rattus rattus*, *R. flavipectus*, and *R. losea* (*2*). Humans can be exposed to hantaviruses by inhaling aerosolized virus from rodent urine or feces or by being bitten by an infected rodent (*3*). SEOV is not known to spread from person to person. 

Rodent-to-rodent transmission occurs through biting or scratching or by exposure from contaminated materials, such as bedding (*4*). Vertical transmission is less likely because rodent progeny are protected by maternal antibodies (*5*). Since 2013, human hantavirus infections transmitted by pet rats have been reported in countries in Europe and the United States (*6*,*7*). In the Netherlands, quantitative reverse transcription PCR (RT-PCR) testing revealed 12.2% positivity among feeder rats not linked to human SEOV cases (*8*). The feeder rodent industry involves cultivating and selling live and frozen rats and mice, primarily as food for reptiles and birds of prey. Although hantavirus infections have occurred in laboratory workers working with rats, little information has existed about hantaviruses in workers at feeder rodent breeding farms (*9*). 

In October 2022, hantavirus infection in a man in his 30s (case-patient A) was reported to the Taiwan Centers for Disease Control (TCDC; Taipei, Taiwan); diagnosis was confirmed by a 4-fold increase in serum IgG from acute- to convalescent-phase serum samples. Initially, the patient manifested fever, generalized malaise, and clinically confirmed coagulopathy and acute renal failure. Upon learning that he worked at a feeder rodent breeding farm, our team sought source and other epidemiologic data to help prevent disease transmission. Because our outbreak investigation involved a notifiable disease, the study was exempt from institutional review board approval. We obtained approval from the Institutional Animal Care and Use Committee at the National Taiwan University College of Medicine and College of Public Health (approval no. 20220344). 

## The Study

**Figure 1:**
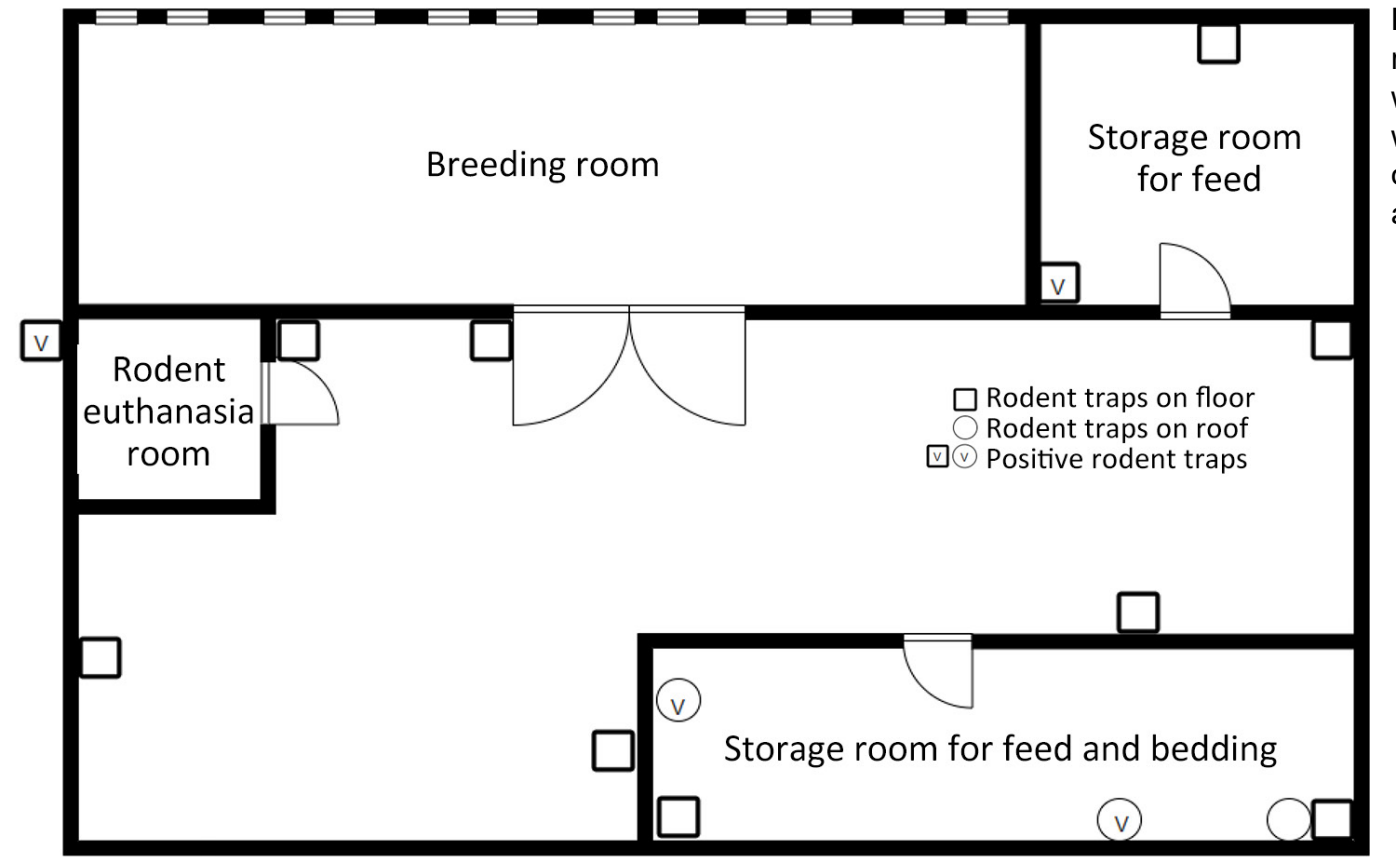
Floor plan of feeder rodent breeding farm and locations where positive feeder rodents were captured in study of recurrent occupational hantavirus infections at the farm, Taiwan.

**Figure 2:**
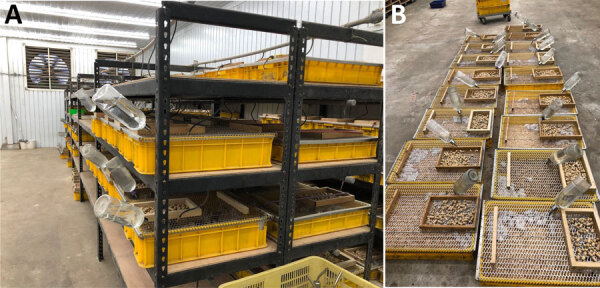
Racks (A) and tubs (B) used at a rodent breeding farm linked to study of recurrent occupational hantavirus infections, Taiwan.

At the time of disease diagnosis, the farm employed 5 workers (including case-patient A) and had ≈12,000 feeder mice (*Mus musculus*) and 2,200 feeder rats (*R. norvegicus*) (Appendix). The owner had introduced no new rodents since 2017. The breeding room in the farm building was an enclosed space (Figure 1). Feeder mice and rats were housed in different racks (Figure 2). Workers were required to wear face masks and gloves when working, but no protocol existed for hand hygiene.

We trapped wild rodents during November 5–9, 2022, ≈18 days after the initial case-patient was reported. Fourteen traps around the farm captured 5 wild rats, all *R. tanezumi*. We also randomly tested 40 feeder mice and 8 feeder rats (Appendix); 3 feeder *R. norvegicus* rats (37.5%) tested positive for hantavirus IgG, whereas all the feeder mice and wild rats tested negative (Table). The owner agreed to humane killing of all feeder rats on the farm on November 19, but the mice colony was not eradicated, and unused bedding and feed were retained. The rat colony was not replenished until February 2023, after later diagnosis of a second worker, case-patient B. 

**Table:** Results of diagnostic testing of rodents for Seoul virus, by date trapped, in study of recurrent occupational hantavirus infections linked to feeder rodent breeding farm, Taiwan *

Species	No. IgG-positive/no. tested (%)	
	2022 Nov 5–9	2023 Feb 11–13
Feeder rodents		
*Mus musculus*	0/40 (0)	NA
*Rattus norvegicus*	3/8 (37.5)	NA
Wild rodents		
*Rattus tanezumi*	0/5 (0)	NA
*Rattus norvegicus*	NA	1/3 (33.3)
*Suncus murinus*	NA	0/5 (0)

*NA, not applicable.

The other 4 workers on the farm reported no hantavirus symptoms during interviews. We also collected and tested blood samples. On the basis of IgM and IgG findings, previous infection was indicated in 1 worker; results for other workers were negative. 

We began wild rodent control using rodenticide and traps on November 9. The rack and floor were disinfected with 5,000 ppm bleach; rat tubs were washed, disinfected, and stored. The enclosed breeder room showed no signs of wild rat activity. Cracks noted in storage rooms for rat feed and bedding were sealed. Thereafter, the owner used bleach to clean used rodent tubs (5,000 ppm) and the racks and floor (1,000 ppm). 

On January 15, 2023, fever and retro-orbital pain developed in case-patient B, who worked at the farm but had tested negative in November 2022. Blood tests revealed thrombocytopenia and elevated liver enzyme levels. We diagnosed case-patient B with hantavirus infection on the basis of a positive IgM titer and a 4-fold rise in IgG titer from acute- to convalescent-phase serum samples. RT-PCR showed hantavirus RNA in her blood. Genomic sequencing data submitted to GenBank (accession no. OR734632) matched with the SEOV nucleoprotein gene. 

The farm had ≈12,000 feeder mice and no feeder rats when the second investigation began on February 10, 2023. We placed 91 traps around the farm and neighborhood and captured 8 wild rats; 1 *R. norvegicus* rat tested positive for hantavirus IgG. We conducted wild rodent control in the neighborhood and discussed with the farm owner the importance of hand hygiene measures and using personal protective equipment (PPE).

## Conclusions

We identified recurrent hantavirus infections in 2 workers on a feeder rodent breeding farm, even though all feeder rats were humanely killed after the first case had been reported. Previous laboratory studies showed that rodents can transmit the virus horizontally through infected bedding (*5*). Because the colony received no additional rodents from 2017 through the end of our testing, the breeder room is enclosed, and we found no signs of wild rat activity, we suspect that the virus was introduced to the breeder colony by infected bedding or feed and then spread through biting and scratching. 

The duration between last exposure and onset of symptoms in case-patient B was 8 weeks. Given incubation for SEOV infection can take up to 8 weeks, case-patient B might have become infected before the infected feeder rats were killed but not diagnosed because of the long incubation period. However, we cannot exclude possible infection from wild rodents or remaining contaminated bedding and feed (*2*). 

Hantavirus infection has been associated with occupational exposure. Several high-risk occupational fields have been identified, including agriculture, forestry, biology fieldwork, and laboratory work (*10*,*11*). Wearing appropriate PPE is crucial because of potentially severe or fatal outcomes. According to the US Office of Animal Care and Use of the National Institutes of Health, rodent facilities should classify their activities as low, moderate, or high risk, and workers should match appropriate PPE use with risk level (*12*). Rodent breeding farms should regularly monitor colony animals for hantavirus infection and maintain ongoing pest control to minimize presence of wild rodents. Staff should wear gloves when handling live or frozen rodents, used bedding, and soiled cages. Proper handwashing should promptly follow glove removal. In a zoonotic outbreak, personnel should wear PPE such as respirators, gloves, washable coveralls, and appropriate footwear. Regular disinfection with bleach or other commercial disinfectants that can effectively kill enveloped viruses is also crucial to prevent environmental contamination (*13*).

HFRS symptoms include fever, headache, muscle aches, and abdominal pain. One study in the United States showed that only 41% of hantavirus case-patients developed an acute illness (*14*). HFRS can manifest in various nonspecific ways, so knowledge of potential animal or environmental exposure is crucial for identifying hantavirus disease in workers. 

Among limitations of this study, we did not test feeder rat organ tissues for hantavirus with RT-PCR, so we were unable to compare viral sequences from case-patients and rats. Also, because we did not test environmental samples, such as bedding and feed, we obtained no direct evidence of virus origin. Finally, we did not test feeder rodents after case-patient B was diagnosed. 

In summary, our findings emphasize that rodent breeding facilities should implement preventive measures such as disinfection protocols and use of PPE. Those actions would lower risk for hantavirus infections among persons working around infected rodents. 

Appendix Additional information from study of occupational hantavirus infections linked to feeder rodent breeding farm.
