# Supplementary material for: Recurrent Occupational Hantavirus Infections Linked to Feeder Rodent Breeding Farm, Taiwan, 2022
Source: Emerg Infect Dis. 2024 Aug;30(8):1702–5. doi: 10.3201/eid3008.230875 (PMC11286064; doi:10.3201/eid3008.230875)
Supplement: Appendix — Additional information from study of occupational hantavirus infections linked to feeder rodent breeding farm. [file 23-0875-Techapp-s1.pdf]

Article DOI: https://doi.org/10.3201/eid3008.230875

EID cannot ensure accessibility for Appendix materials supplied by authors. Readers who have difficulty accessing Appendix content should contact the authors for assistance.

Recurrent Occupational Hantavirus Infections Linked to Feeder Rodent Breeding Farm, Taiwan, 2022

Appendix

Farm Building Layout and Feeder Rodent Processing

The farm building contained a breeding room, euthanization room, and two storage rooms. The breeding room was an enclosed space; all feeder mice and rats were housed in the same room but in different breeding racks (Figure 2). Each rack of rats had up to 20 tubs, each containing 5–7 rats; each rack of mice had 42 tubs, each containing 7–10 mice. At age 2 months the feeder mice/rats were humanely killed with CO_2 and then vacuum-packed and stored in a freezer for sale on the internet.

Rodent Blood Collection Procedure for Hantavirus Testing

Rodents from the lowest tub in each rack were anaesthetized with isoflurane. We collected into a microtube ≈0.5 mL of blood from the hearts of these wild and feeder mice/rats and stored it on ice. We identified rodent species on the spot, and blood samples were transported to the TCDC laboratory. Hantavirus IgM/IgG Dx Select kits (FOCUS Diagnostics, CA, USA) were used for detecting hantavirus IgM/IgG antibodies (*1*).

Sample Size Calculation

$$n = \left[1 - (\alpha)^{\frac{1}{d}}\right](N - \frac{d-1}{2})/s$$

In this formula:

α corresponds to the complement of our chosen confidence level, which was set at 0.05 to achieve a 95% confidence interval. N represents the total population size, which was 12,000 in our case; d signifies the anticipated number of diseased animals within the population, estimated at 10% on the basis of the assumed prevalence of hantavirus (therefore, we assigned a value of 1,200 to d); s denotes the sensitivity of the test, which we estimated at 80%.

On the basis of this formula, the calculated sample size was 36. However, we opted to include a sample of 40 mice in our research.

Reference

1. Koraka P, Avsic-Zupanc T, Osterhaus AD, Groen J. Evaluation of two commercially available immunoassays for the detection of hantavirus antibodies in serum samples. J Clin Virol. 2000;17:189–96. PubMed https://doi.org/10.1016/S1386-6532(00)00096-2
